# The impact of atrial voltage and conduction velocity phenotypes on atrial fibrillation recurrence

**DOI:** 10.3389/fcvm.2024.1427841

**Published:** 2024-12-16

**Authors:** Pedro Silva Cunha, Sérgio Laranjo, Sofia Monteiro, Guilherme Portugal, Cátia Guerra, António Condeixa Rocha, Mariana Pereira, Rui Cruz Ferreira, Jordi Heijman, Mário Martins Oliveira

**Affiliations:** ^1^ Arrhythmology, Pacing and Electrophysiology Unit, Cardiology Service, Santa Marta Hospital, Lisbon, Portugal; ^2^ Centro Clínico Académico, Hospital de Santa Marta, Lisboa, Portugal; ^3^ Physiology Institute, Faculdade de Medicina, University of Lisbon, Lisbon, Portugal; ^4^ CCUL @ RISE, Faculdade de Medicina, University of Lisbon, Lisbon, Portugal; ^5^ Comprehensive Health Research Center, NOVA Medical School, Faculdade de Ciências Médicas, NMS, FCM, Universidade NOVA de Lisboa, Lisboa, Portugal; ^6^ Departamento de Fisiologia, NOVA Medical School, Faculdade de Ciências Médicas, NMS, FCM, Universidade NOVA de Lisboa, Lisboa, Portugal; ^7^ Instituto de Telecomunicações, Instituto Superior Técnico, Lisbon, Portugal; ^8^ Electrophysiology, Biosense Webster, Lisbon, Portugal; ^9^ Department of Cardiology, Cardiovascular Research Institute Maastricht, Maastricht University, Maastricht, Netherlands; ^10^ Gottfried Schatz Research Center, Division of Medical Physics & Biophysics, Medical University of Graz, Graz, Austria

**Keywords:** atrial fibrillation, atrial conduction velocity, voltage, atrial myopathy, ablation & electrophysiology

## Abstract

**Introduction:**

Low atrial voltage and slow conduction velocity (CV) have been associated with atrial fibrillation (AF); however, their interaction and relative importance as early disease markers remain incompletely understood. We aimed to elucidate the relationship between atrial voltage and CV using high-density electroanatomic (HDE) maps of patients with AF.

**Methods:**

HDE maps obtained during sinus rhythm in 52 patients with AF and five healthy controls were analysed. Atrial voltage and CV maps were generated, and their correlations were assessed. Subgroup analyses were performed based on clinically relevant factors such as AF type, CV, and voltage levels. Finally, cluster analysis was conducted to identify distinct phenotypes within the population, reflecting different patterns of conduction and voltage.

**Results:**

A moderate positive correlation was found between the mean atrial voltage and CV (*r* = 0.570). Subgroup analysis revealed differences in voltage (*p* = 0.0044) but not in global CV (*p* = 0.42), with no significant differences between AF types. Three distinct phenotypes emerged: normal voltage/normal CV, normal voltage/low CV, and low voltage/low CV, with distinct recurrence rates, suggesting different disease progression paths. Slower atrial CV was identified as a significant predictor of arrhythmia recurrence at 12 and 24 months after AF ablation, surpassing the predictive potential of atrial voltage.

**Conclusion:**

Atrial voltage and CV analyses revealed distinct phenotypes. Lower atrial CV emerged as a significant predictor of AF recurrence, exceeding the predictive significance of atrial voltage. These findings emphasise the importance of considering CV and voltage in managing AF and offer potential insights for personalised strategies.

## 1 Introduction


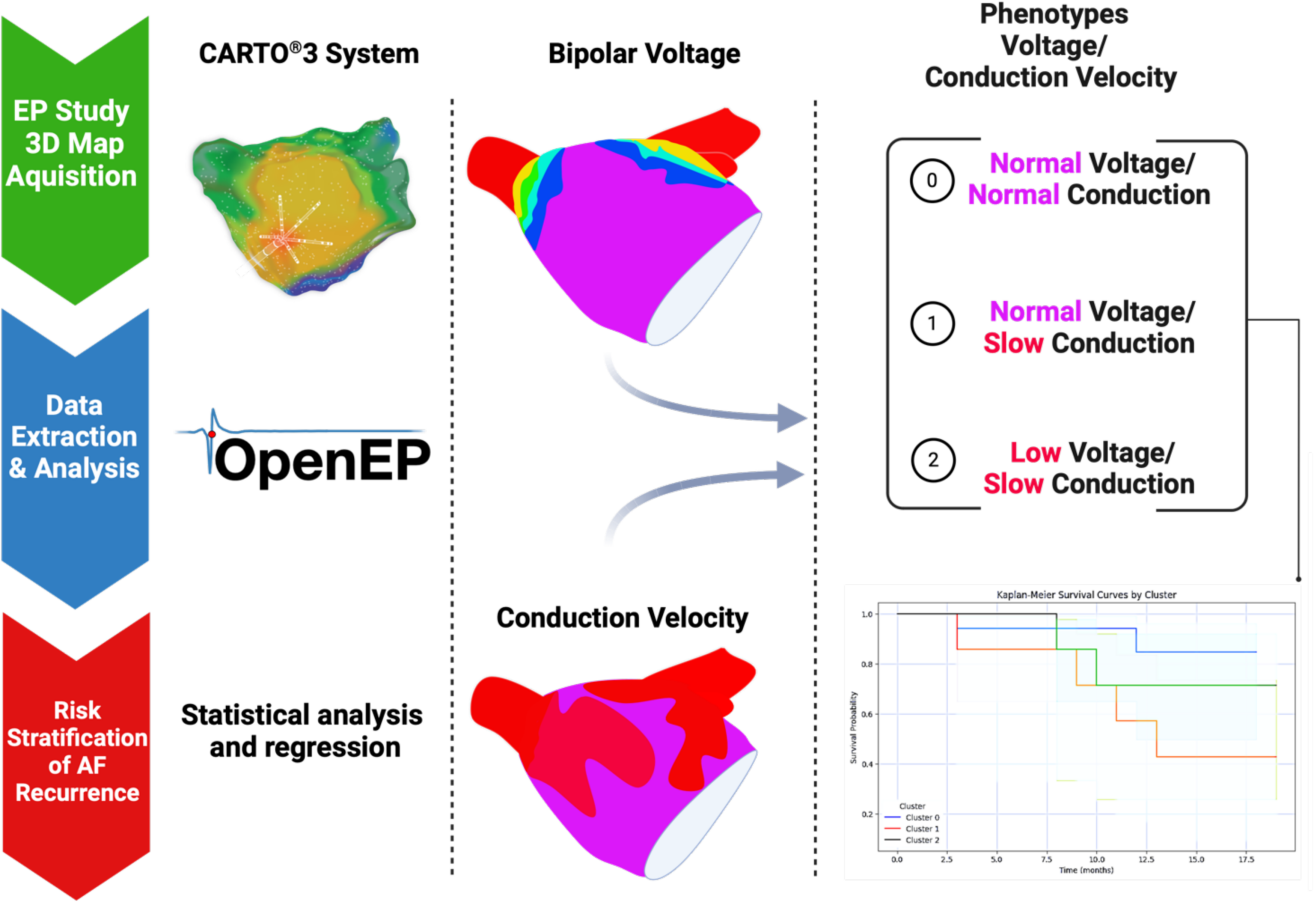


Atrial fibrillation (AF) is a prevalent cardiac arrhythmia affecting millions of people globally (1, 2). Its incidence is projected to rise significantly, given the ageing demographics and advancements in screening and diagnostics (3). AF poses a substantial healthcare burden, contributing prominently to heightened morbidity and mortality, partially stemming from stroke, heart failure, and other cardiovascular complications (4–6). Therefore, early recognition and intervention are imperative for effective patient management and improved outcomes. The pathophysiology of AF is intricate and multifaceted (7), characterised by both electrical and structural remodelling of the atria, creating a substrate conducive to the persistence and progression of arrhythmia (8, 9). Structural remodelling encompasses atrial dilation and fibrosis, whereas electrical remodelling involves alterations in ion channel function, intracellular calcium handling, and gap-junction remodelling (10). These remodelling processes collectively reduce atrial voltage and induce changes in conduction velocity (CV), which are detectable using electroanatomic mapping techniques (11).

Despite prior investigations into the role of atrial voltage and CV in AF (12, 13), the correlation between these parameters and their link to disease progression remains unknown. Our study aimed to examine the relationship between atrial voltage and CV in patients with AF using high-density electroanatomic mapping. Our hypothesis posited the existence of a subgroup of patients with dissociation between these parameters, suggesting a potential marker for distinct stages of AF. If validated, this could help improve AF classification, identify high-risk patients for AF progression, enable timely intervention, and optimise overall patient outcomes.

## 2 Methods

### 2.1 Study population and design

In this single-centre retrospective study, we included patients who underwent elective AF ablation using high-density electroanatomic mapping from January 2019 to January 2022. The main objective of this study was to explore the correlation between atrial voltage and CV in patients with AF and assess their potential as early indicators of disease.

We considered adult patients (aged > 18 years) with symptomatic paroxysmal AF (PAF) or persistent AF (PsAF) who were referred for catheter ablation with electroanatomic mapping as part of their clinical management. Only “*de novo*” ablations and cases without previous substrate modification were accepted. AF was classified as PAF if arrhythmia episodes resolved spontaneously or with intervention within seven days of onset or as PsAF if episodes lasted longer than seven days but less than 12 months.

The exclusion criteria were: (a) contraindications to catheter ablation, such as active infection, coagulopathy, or severe comorbidities that would make the procedure unfeasible; (b) previous catheter ablation for AF or any other atrial arrhythmia, as the procedure could potentially modify the atrial substrate and thus skew the analyses; (c) significant structural heart disease, including moderate-to-severe valvular disease, congenital heart disease, systolic heart failure, or a history of myocardial infarction, as these conditions could also alter the atrial substrate and influence the relationship between atrial voltage and conduction velocity; and (d) insufficient number of mapping points (< 1,000 points) in the left atrium (LA). Only maps acquired in sinus rhythm with more than 1,000 reading points and well-distributed anatomical coverage were included.

We also included a control group of five patients presenting with a left-sided accessory pathway scheduled for elective catheter ablation (as a first procedure), none of whom had a history of documented AF (by ECGs and Holter recordings).

Baseline demographic, clinical, and echocardiographic data were collected from hospital electronic medical records for all patients. These included age, sex, body-mass index, AF type (paroxysmal or persistent), AF duration, comorbidities, medication, LA diameter, and left ventricular ejection fraction (LVEF).

All participants provided written informed consent for the use of their anonymised data. The Ethics Committee of Santa Marta Hospital, Lisbon approved the study protocol.

### 2.2 Ablation procedure

Our approach has been described elsewhere (14). Briefly, every patient underwent a routine preprocedural transthoracic echocardiogram to evaluate LVEF and LA dimensions and to screen for structural heart disease, as well as cardiac computed tomography or magnetic resonance imaging (with segmentation of the LA) to assess anatomy and to exclude the presence of intracardiac thrombi. Additionally, if the imaging study was performed > 48 h before ablation, transoesophageal echocardiography was performed on the day of the procedure (to exclude thrombi). Patients underwent ablation after continued oral anticoagulation (for at least four weeks) using warfarin with a therapeutic INR (2.0–3.0) or direct oral anticoagulants (DOAC), with one dosage omitted before the ablation. All antiarrhythmic drugs were discontinued for at least five half-lives before ablation. Continuous monitoring of oxygen saturation and ECG was maintained throughout the ablation. All procedures were performed under conscious sedation. The ablation strategy for all patients consisted of antral isolation of all pulmonary veins (PV) using an open-irrigated-tip radiofrequency ablation catheter (ThermoCool SmartTouch® SurroundFlow, Biosense Webster) with point-by-point lesions. No additional lines were performed.

Electroanatomical mapping was performed using the Carto® 3 System version 7 (Biosense Webster Inc., Diamond Bar, CA, USA), enabling visualisation of the cardiac chambers and real-time assessment of electrical activity within the atria based on a combination of magnetic and impedance-based technologies to localise the catheter within the heart. We used the 20-pole PentaRay (Biosense-Webster Inc., Diamond Bar, CA), a steerable catheter with 180° unidirectional flexion. This catheter features 1 mm electrodes distributed over five soft, radiating spines, with an interelectrode spacing of 2–6–2 mm, for high-density mapping of the LA. A minimum of 1,000 points per atrium were collected, ensuring a high-density map for each patient. Real-time display of the acquired electrogram data on the Carto® 3 system enabled the operator to assess data quality and make necessary adjustments. The map points were collected only if the Tissue Proximity Indicator (TPI) was fulfilled. TPI is a crucial feature for ensuring accurate voltage data collection. The system does not collect voltage points unless the TPI confirms adequate catheter-tissue contact. This ensures that the recorded points reflect true tissue proximity and are not artefacts from poor or inconsistent contact. The CARTO system uses a combination of impedance, magnetic field-based localisation, and contact force measurements (depending on the catheter used) to confirm that the catheter is in stable contact with the tissue. When the TPI requirements are met, the system registers the point, ensuring accurate voltage readings for constructing the 3D map. This mechanism enhances the precision of the mapping process and avoids the inclusion of points where the catheter might not have proper contact with the atrial wall. This approach minimises the likelihood of erroneous data due to suboptimal contact and provides reliable information for analysis. Given this, all points in the voltage map inherently have adequate TPI values, ensuring high-quality data for interpretation.

Pulmonary vein isolation was confirmed by performing systematic pacing manoeuvres to confirm entry and exit block.

### 2.3 Post-ablation evaluation

AF recurrence-free survival was the primary endpoint, considering an AF event as any post–90–day blanking atrial tachyarrhythmias lasting 30 s or longer, sustained symptomatic episodes of rapid palpitations, new prescription of class I or III antiarrhythmic drugs (AAD) or repeated ablation. After the ablation procedure, the patients were discharged on AAD at the operator's discretion, together with oral anticoagulation. They were observed for routine follow-up in the outpatient clinic 1–3 months after the procedure and every six months (or earlier if symptoms were present) during the first two years post-ablation. A standard 12-lead ECG was obtained at each visit. After the blanking period, patients were followed up with a 24-h Holter at each outpatient visit. The AAD was discontinued six months after the ablation procedure and withdrawn if the patients were free from arrhythmia-related symptoms. Oral anticoagulation was reevaluated in the third month, and the decision to continue was based on the CHA_2_DS_2_-VASc score. Clinical events that occurred during the follow-up period were evaluated.

### 2.4 Data pre-processing and analysis

The electroanatomic mapping data acquired during the procedures were exported from the Carto® 3 System for further analysis using the OpenEP software. OpenEP, developed by the CEMRG group at King's College London, is a MATLAB-based platform for analysing cardiac electrophysiology data (15). The software provides a comprehensive suite of tools for pre-processing, analysing, and visualising electroanatomic mapping data. Data preprocessing included several steps to ensure the quality and consistency of the data for subsequent analysis, as follows:

 *Noise reduction*: The software excluded electrograms with high-frequency noise or artefacts that could interfere with the analysis.
 *Filtering:* The electrograms were subjected to bandpass filtering in the 30–250 Hz frequency range to remove any baseline-wander and high-frequency noise while preserving the frequency content of interest in the electrograms and allowing for more accurate annotation of local activation times (LATs).
 *Annotation:* LATs were automatically annotated for each electrogram using a customised peak detection algorithm provided by the OpenEP software. The algorithm identified the steepest downslope of the bipolar electrogram as the local activation time, ensuring a consistent and objective approach for annotation across all patients.

After preprocessing, the PV and LA appendage were semi-automatically cut from the LA geometry (16) using the method described by Tobon-Gomez et al. (17). The OpenEP software was then used to generate atrial voltage and CV maps for each patient.

### 2.5 Voltage-map signal analysis

**Figure 1:**
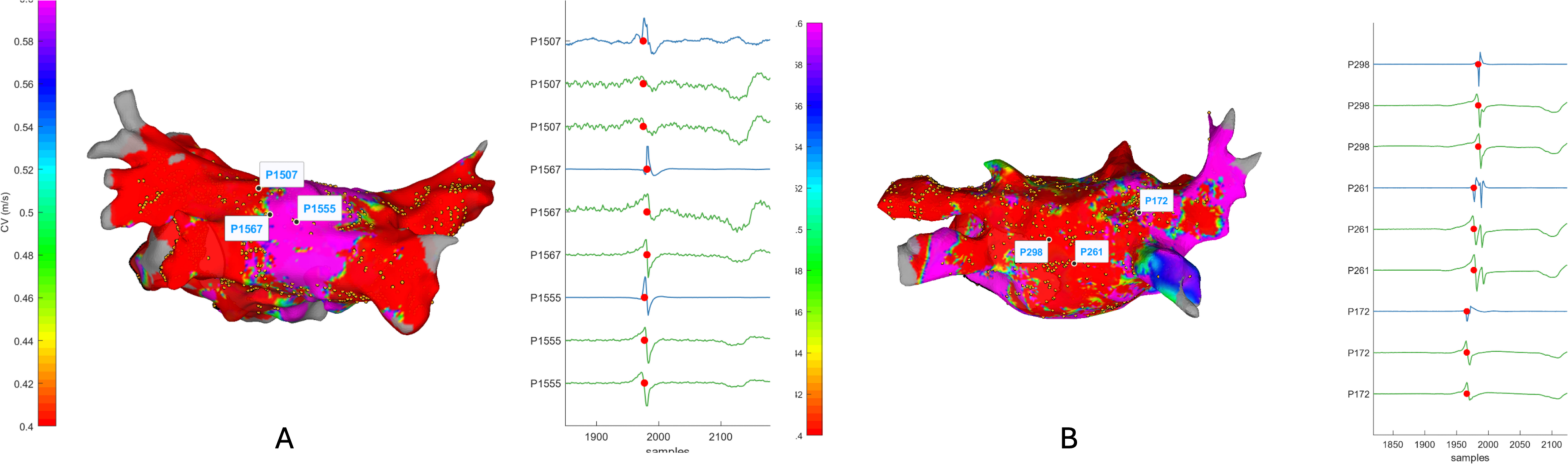
Electrogram display/annotation. OpenEP measurement of local electrical activity. Examples of electrogram annotation: Panel **(A)**, normal conduction; Panel **(B)** slow conduction. Blue lines: bipolar electrograms; Green lines: unipolar electrograms.

Voltage maps were created based on bipolar electrograms recorded from the atrial endocardial surface during the electroanatomic mapping. The critical parameter derived from electrograms is the peak-to-peak amplitude, which reflects the atrial voltage at each mapping point. The software calculated this amplitude by identifying the maximum and minimum voltage values within a predefined time window and determining the absolute difference between these values. After calculating the peak-to-peak amplitudes for each electrogram, the software interpolates the voltage data onto the 3D atrial geometry, producing a voltage map that presents the spatial distribution of the atrial voltages. Radial basis function interpolation, such as inverse distance weighting, was employed to estimate the voltage values at points between mapping points and provide a smooth, continuous representation of the atrial voltage. Colour-coded voltage maps facilitate atrial substrate visualisation, displaying high-voltage regions in shades of blue or purple and low-voltage areas (LVAs) in red or orange (Figure 1). LVAs, an accepted indicator of fibrotic tissue, were defined as areas with a bipolar peak-to-peak voltage <0.50 mV. On the voltage map, the bipolar voltage colour bar ranged from 0.10 to 0.50 mV.

### 2.6 Conduction velocity map signal analysis

Estimating CV from electroanatomic mapping data involves analysing LATs and the spatial distribution of mapping points. The steepest downslope of the bipolar electrogram, representing the point of the maximum depolarization rate, typically annotates LATs during the pre-processing stage. OpenEP software uses a previously validated algorithm (15) that considers wavefront curvature and activation direction to compute the CV. Although electroanatomic mapping systems provide detailed representations of electrophysiological data, it is essential to access the individual electrograms (raw electrogram data) used to create the map to analyse electrogram characteristics (Figure 1).

The triangulation algorithm included in OpenEP was used to estimate the CV from a set of LAT measurement locations. Triplets of the electrodes were selected automatically through Delaunay triangulation. Constraints were imposed on the electrode distance (between 1.5 and 2.0 mm), the difference in activation times between vertices (larger than 2 ms), the minimum line of block CV (0.2 m/s), and the minimum angle allowed within a triangle (30°) (18).

The algorithm calculates the LATs' spatial gradients across neighbouring mapping points and the distance between points, estimating the electrical propagation velocity at each location.

**Figure 2:**
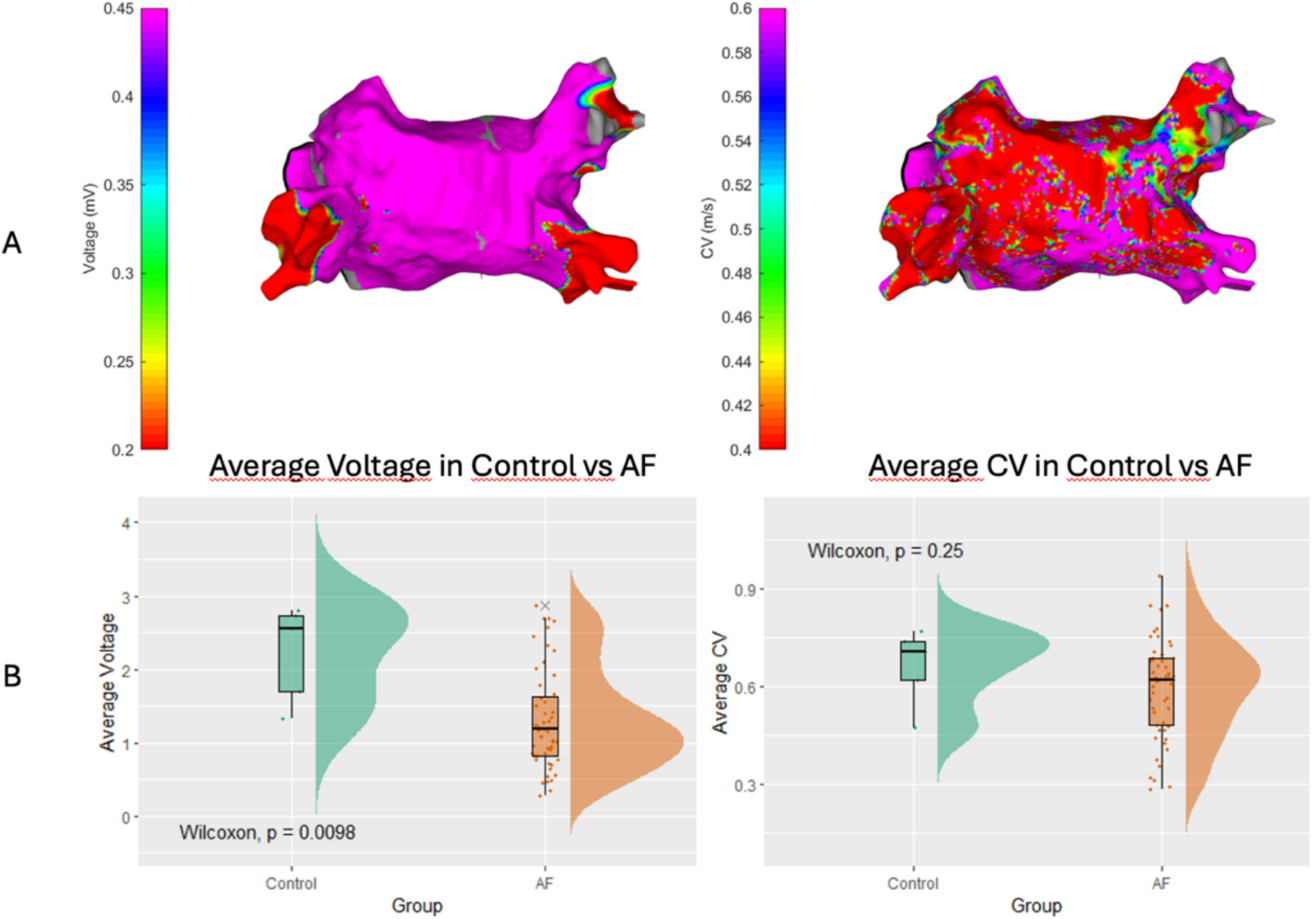
Left atrial voltage and conduction velocity in control and AF groups. Panel **(A)** Examples of colour-coded maps for atrial voltage (left) and conduction velocity (right). Panel **(B)** Average voltage (left) and average conduction velocity (right) in control and AF patients.

The CVs were subsequently interpolated onto 3D atrial geometry to generate a map of the spatial distribution of the CV. Like voltage maps, CV maps are colour-coded with fast conduction in blue or purple and slow conduction in red or orange (Figure 2). These maps help to identify areas contributing to arrhythmogenesis due to slow conduction or sudden changes in CV, which might suggest the presence of conduction barriers or functional blocks.

### 2.7 Statistical analysis

Statistical analyses were conducted using R 4.2.2 software (R Core Team 2021. R: A language and environment for statistical computing. R Foundation for Statistical Computing, Vienna, Austria) complemented by Python for clustering and visualisation. Continuous variables, such as atrial voltage and CV, were reported as means with standard deviations (SD) or medians with interquartile ranges (IQR), depending on their distribution. The normality of the dataset was assessed using the Shapiro-Wilk test alongside visual inspections of histograms and Q-Q plots. Spearman's rank correlation coefficient (rho) was utilised to evaluate the association between atrial voltage and CV. It was chosen for its robustness against non-linear relationships and effectiveness in identifying monotonic associations between variables. Categorical variables were described using frequencies and percentages, providing an overview of the cohort composition.

The study cohort was stratified into three CV categories based on the following criteria: slow CV (<0.6 m/s), moderate CV (>0.6 m/s and <0.8 m/s), and high CV (>0.8 m/s). Similarly, the LA voltage was classified into three ranges: low (<0.5 mV), intermediate (0.5–0.75 mV), and normal (0.75–1.0 mV). We employed one-way ANOVA when the data conformed to a normal distribution for comparative analysis of the average LA voltage and CV. Conversely, in cases where the data deviated from the normal distribution, we used the Kruskal-Wallis test. Furthermore, we extended our analysis to compare LA CV (A) and voltage (B) between the control group and patients with AF. Given the nature of the data, the Wilcoxon test was used for this comparison.

K-means clustering was applied to identify patterns within the CV groups. This approach facilitated categorising patients into distinct clusters based on the similarities in their CV and voltage profiles. The clusters were characterised and analysed using descriptive statistics.

Survival analysis determined the time until AF recurrence, considering recurrence as the event of interest. A Kaplan-Meier estimator was initially used to obtain recurrence-free survival estimates. Log-rank tests were then applied to compare AF survival functions between the subgroups.

All statistical tests were two-sided, and the significance level was *p* < 0.05. The methodology and reporting of our findings were aligned with the guidelines of the Strengthening the Reporting of Observational Studies in Epidemiology (STROBE) statement (19).

## 3 Results

### 3.1 Clinical characteristics

Our cohort comprised 52 patients with AF: 61.5% (*n* = 32) had PAF and 30.8% (*n* = 16) were female (Table 1). AF recurrence at 24 months follow-up was observed in 36.5% (*n* = 19) of patients. Comorbidities were common, with 67.3% (*n* = 35) of the patients having systemic hypertension, 30.8% (*n* = 16) having hyperlipidaemia, and 11.5% (*n* = 6) having diabetes mellitus. Additionally, 15.4% (*n* = 8) of the patients were smokers, 7.7% (*n* = 4) had obstructive sleep apnoea syndrome (OSAS), and 13.5% (*n* = 7) had LVEF <40%. The distribution of comorbidities varied, with 15.4% (*n* = 8) of patients having no comorbidities, 26.9% (*n* = 14) having one, 25.0% (*n* = 13) having two, 23.1% (*n* = 12) having three, and the remainder having four or more comorbidities (Table 1). The control group consisted of five patients (two female; 42.0 ± 14.8 years) without a history of AF or comorbidities who underwent a cardiac electrophysiology study for a left atrial accessory pathway in the same period.

**Table 1:** Baseline patient characteristics (*N* = 52).

Baseline characteristics	
Age (years)	56.9 ± 12.2
BMI (kg/m^2^)	28.4 ± 4.0
LAVI (ml/m^2^)	36.0 ± 9.7
Ejection fraction (%)	56.0 ± 9.7
AF type	
Paroxysmal	32 (61.5)
Persistent	20 (38.5)
Female Sex	16 (30.8)
Hypertension	35 (67.3)
Hyperlipidemia	16 (30.8)
Diabetes mellitus	6 (11.5)
OSAS	4 (7.7)
Smoking	8 (15.4)
COPD	1 (1.9)
Chronic kidney disease	1 (1.9)
Thyroid disease	8 (15.4)
Cancer	3 (5.8)
Coronary disease	3 (5.8)
Vascular disease	3 (5.8)
Valvular disease	1 (1.9)
Previous stroke	2 (3.8)
Thromboembolism	0 (0)
Hypertrophic cardiomyopathy	1 (1.9)
Nr comorbidities	
0	8 (15.4)
1	14 (26.9)
2	13 (25)
3	12 (23.1)
4	3 (5.8)
5	2 (3.8)
CHA2DS2_VASc score	
0	12 (23.1)
1	10 (19.2)
2	16 (30.8)
3	7 (13.5)
4	7 (13.5)
Average voltage	1.18 (0.82–1.63)
Average unipolar voltage	1.92 (1.37–2.44)
Average CV	0.62 (0.48–0.69)
Area Bip 05_rel	0.23 (0.15–0.32)
Area Bip 03_rel	0.17 (0.09–0.23)
Area CV 06_rel	0.36 (0.29–0.46)
Area CV 04_rel	0.17 (0.13–0.25)
Number of points collected (3D map)	1,654.2 ± 874.5
Recurrence (%)	19 (36.5)
Follow up time	18.9 ± 10.8

Values mean ± SD, *n* (%), or median (IQR).

AF, atrial fibrillation; LA, left atrium; OSAS, obstructive sleep apnoea syndrome; COPD, chronic obstructive pulmonary disease; BMI, body mass index; LAVI, left atrial volume Index; Area Bip, bipolar voltage area, with cut-off <0.5 and 0.3 mV; Area CV, conduction velocity area, with cut-off <0.6 and 0.4 m/s.

### 3.2 Comparison of controls and patients with AF

Our comparative analysis between control subjects and patients with AF showed statistically significant differences in LA voltage and CV, as depicted in Figure 2B. The control group exhibited an average LA voltage of 2.22 ± 0.66 mV vs. 1.3 ± 0.68 mV in the AF patient group (*p* = 0.0098). The average CV in control subjects (0.66 ± 0.12 m/s) was numerically faster than in AF patients, with an average CV of 0.59 ± 0.15 m/s, but this did not reach statistical significance (*p* = 0.25; Figure 2B).

### 3.3 Correlation between conduction velocity and voltage

**Figure 3:**
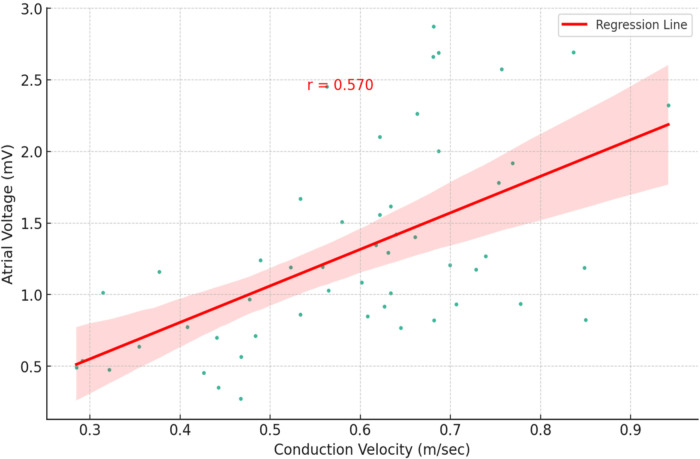
Correlation between atrial voltage and conduction velocity in this cohort of atrial fibrillation patients. Each point on the plot corresponds to an individual patient, with the *x*-axis depicting conduction velocity (measured in meters per second) and the *y*-axis illustrating atrial voltage (measured in millivolts). The red regression line represents the moderate positive correlation between these two variables, with a correlation coefficient *r* = 0.570.

We identified a moderate positive correlation between global CV and average LA voltage, as depicted in Figure 3 (*r* = 0.570). This correlation suggests a significant relationship wherein a global loss of myocardium, reflected in lower voltage amplitudes, tends to be associated with a decrease in CV.

**Figure 4:**
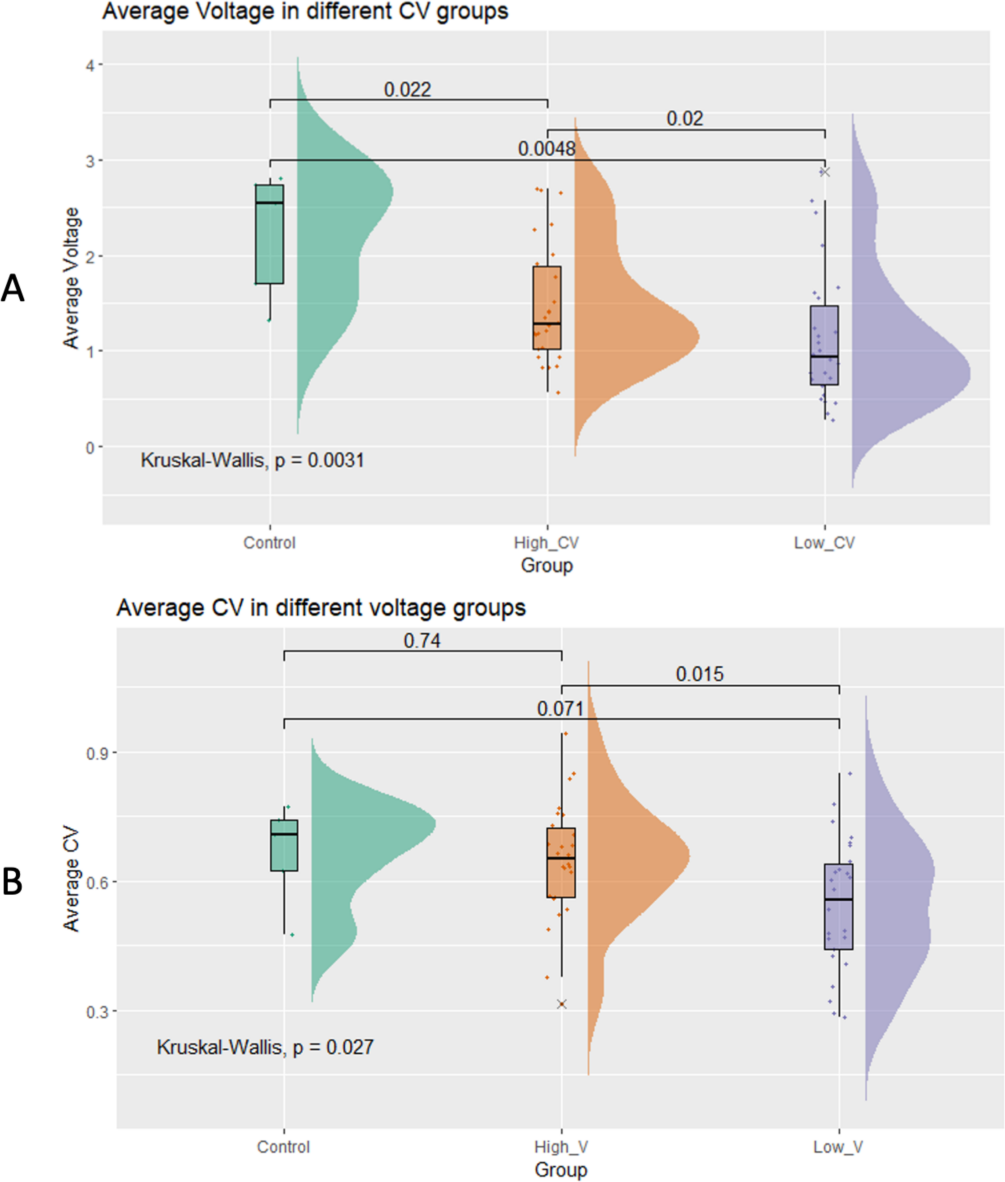
Comparison of the average left atrial voltage **(A)** and conduction velocity **(B)** in different conduction velocity groups.

We subsequently investigated the relationship between CV and LA voltage in patients with AF. Consistent with the generally positive correlation, stratifying AF patients based on CV revealed that those in the high CV category exhibited a notably larger average voltage (1.47 ± 0.62 mV) than those in the low CV group (1.13 ± 0.71 mV; *p* = 0.02; Figure 4A). Similarly, patients with larger LA voltage demonstrated an average CV of 0.64 ± 0.13 m/s vs. 0.54 ± 0.15 m/s observed in the low voltage group (*p* = 0.015; Figure 4B).

### 3.4 Subgroup analysis: AF types

**Figure 5:**
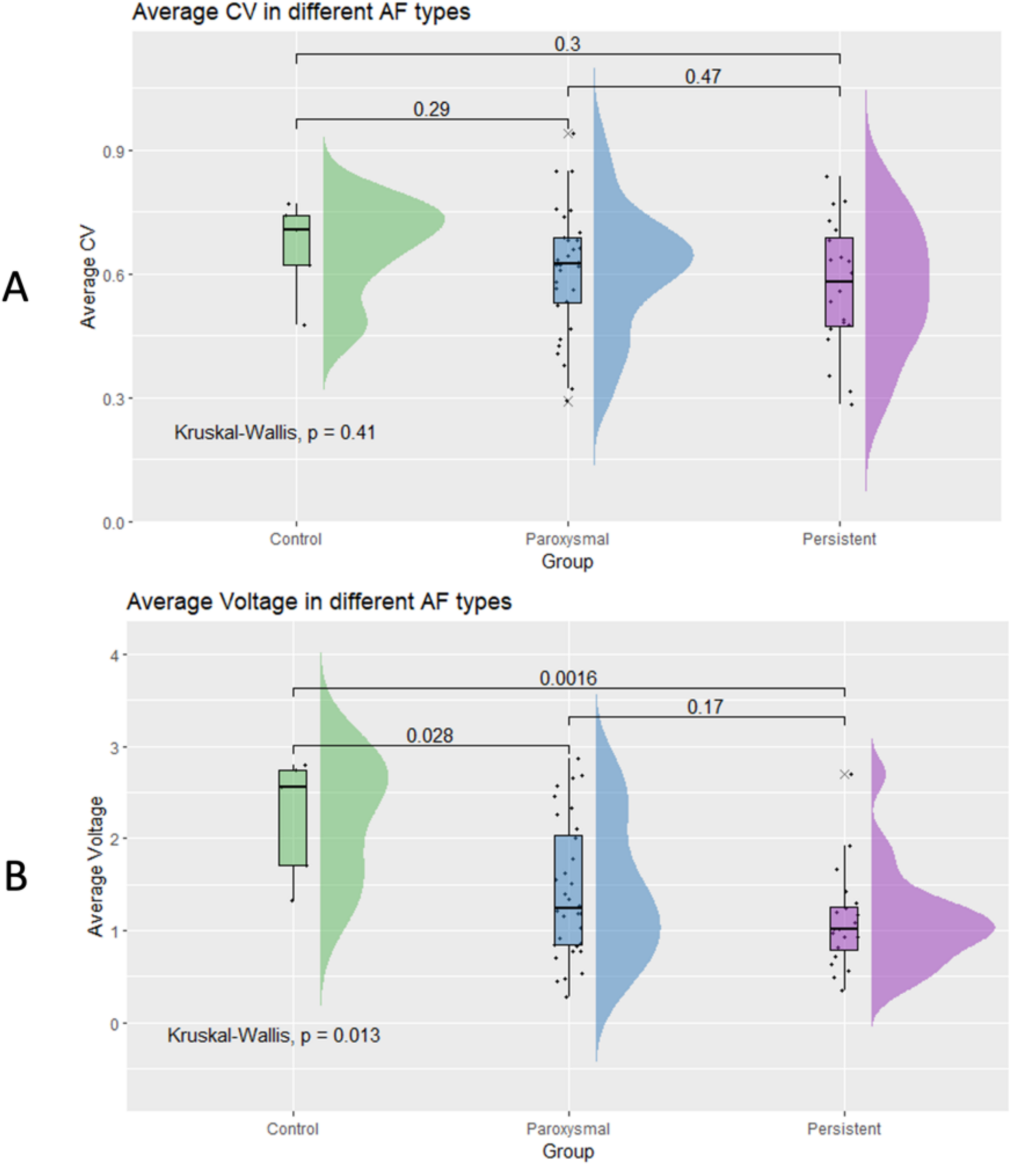
Differences in average conduction velocity **(A)** and average voltage **(B)** between paroxysmal AF patients and persistent AF patients.

Subgroup analysis revealed no significant differences in average CV between PAF and PersAF patients (*p* = 0.47; Figure 5A). The average voltage in control subjects (2.22 ± 0.66 mV) was significantly larger than in either PAF (1.42 ± 0.74 mV) or PersAF patients (1.1 ± 0.53 mV), but there was no significant difference in atrial voltage between PAF and PersAF (*p* = 0.17; Figure 5B).

### 3.5 Identification of distinct phenotypes in AF patients

**Figure 6:**
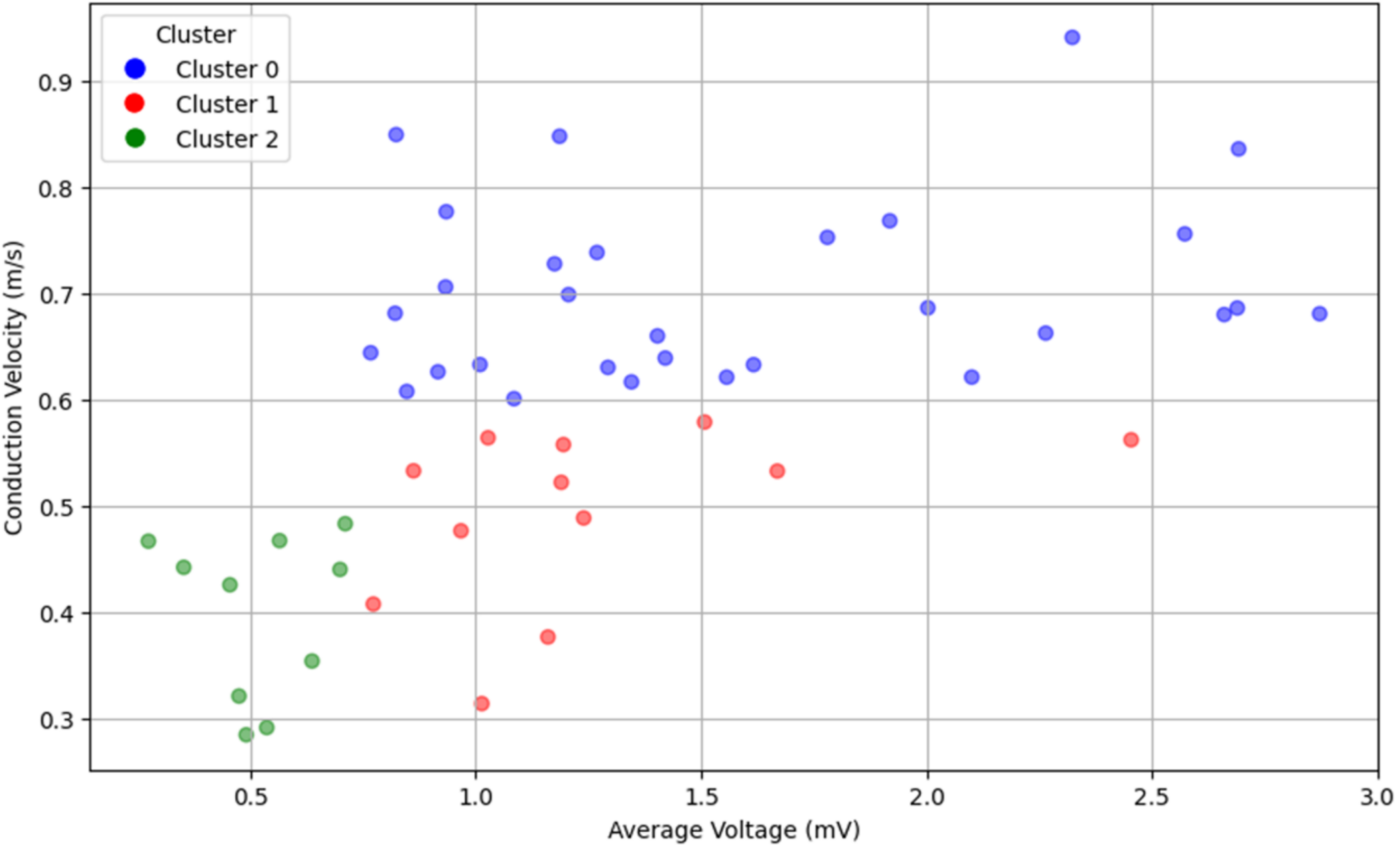
Visualisation of three unique phenotypes based on average conduction velocity and voltage in a cohort of 52 patients with atrial fibrillation. The normal conduction velocity and normal voltage define Cluster 0 (blue). Cluster 1 (red) is characterised by slow conduction velocity and normal voltage. Cluster 2 (green) encompasses slow conduction velocity and low voltage. The plot delineates the heterogeneity and multifaceted nature of AF, reflecting the different patterns of conduction and voltage.

**Figure 7:**
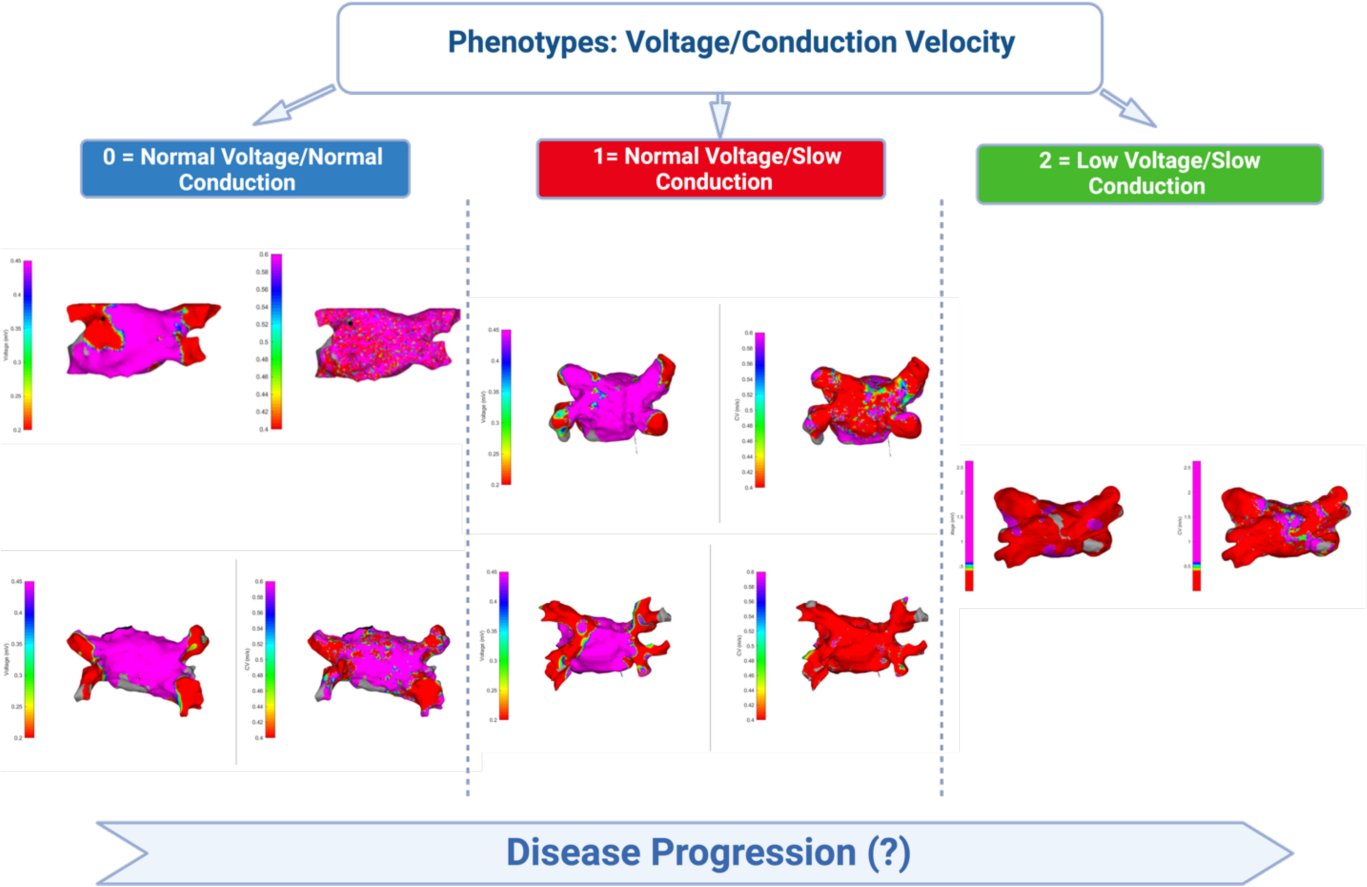
Schematic depiction of the observed phenotypes of the relationship between atrial voltage and atrial conduction velocity. Cluster 0 is characterised by normal mean atrial voltage and normal conduction velocity, Cluster 1 by slow conduction velocity and normal voltage, whereas Cluster 2 encompasses slow conduction velocity and low voltage. In each cluster panel, the image on the left represents the voltage colour map and the image on the right represents the velocity colour map.

Observing a moderate positive correlation between atrial voltage and CV (*r* = 0.570) led us to hypothesise that subgroups may have distinct phenotypes (combinations of CV and voltage). Three different phenotypes (Figures 6, 7), characterised by CV, voltage, and specific clinical characteristics, emerged by analysing them unbiasedly to explore this hypothesis. Thus, they highlight the heterogeneity and multifaceted nature of AF.

Cluster 0: Defined by normal CV and voltage, this cluster encompassed 57.7% of the cohort (*n* = 30). Most patients (66.7%) were diagnosed with PAF, whereas one-third (33.3%) had PersAF. The distribution of comorbidities was diverse, but the comorbidity burden was low, with the majority having only one comorbidity. The mean LA volume was 33.15 ml/m^2^, which similarly reflects less advanced atrial remodelling.

Cluster 1: 23.1% of the cohort (*n* = 12) had this phenotype, which exhibited a slow CV but a normal voltage. The majority (58.3%) had PAF, and 41.7% were diagnosed with PersAF. Recurrence was 33.3%, and the comorbidity distribution was 25% with no comorbidities and 41.7% with two. The mean LA volume was 39.25 ml/m^2^.

Cluster 2: Comprising 19.2% of the cohort (*n* = 10), this cluster was marked by low voltage and slow CV. The AF type was evenly divided between PAF and PersAF (50% each), and recurrence was observed in 60% of the patients. Notably, half of the patients in this cluster had three comorbidities. The mean LA volume was 40.40 ml/m^2^, suggesting more pronounced alterations in the atrial tissue.

### 3.6 Survival analysis

The global cohort AF recurrence rate was 30%. We subsequently analysed the arrhythmic recurrence per cluster. Cluster 2 (low voltage/slow conduction) presents the worse prognosis, with a recurrence rate of 60%. Nevertheless, cluster 1 (normal voltage/slow conduction), compared to the group of patients with normal voltage and normal CV (Log-rank test Cluster 0 vs. Cluster 1: *p*-value = 0.0468), shows a significant difference in arrhythmic recurrence. This means that although we observe a normal voltage, we are not necessarily covering the entire substrate disease spectrum, and CV adds prognostic value.

**Figure 8:**
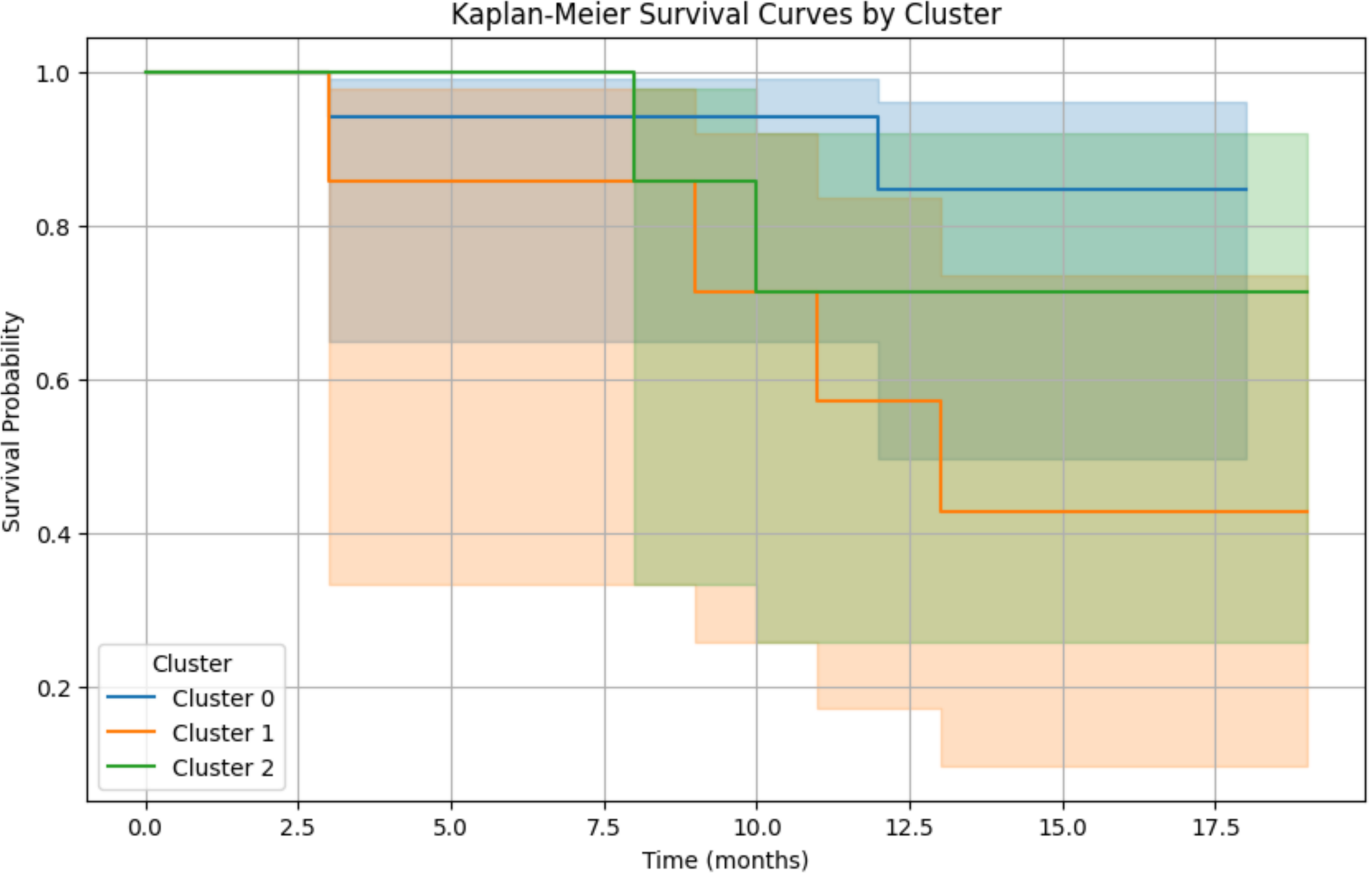
Arrhythmia recurrence according to cluster, kaplan-meier estimates recurrence-free survival*.* Log-rank test Cluster 0 vs. Cluster 1: *p*-value = 0.0468. Log-rank test Cluster 0 vs. Cluster 2: *p*-value = 0.3990. Log-rank test Cluster 1 vs. Cluster 2: *p*-value = 0.4027.

**Figure 9:**
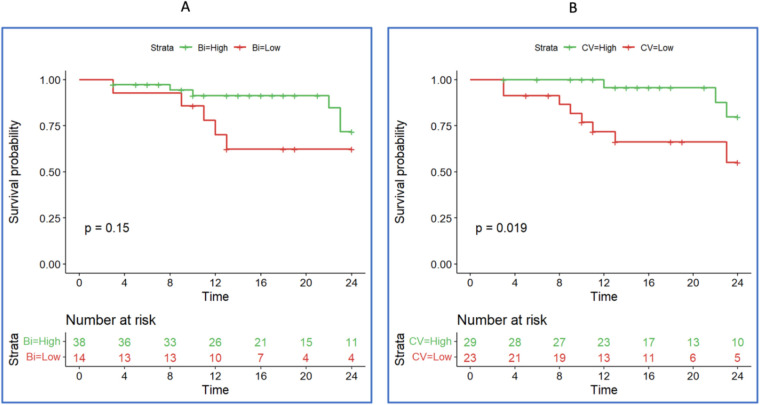
According to atrial voltage (panel **A**) and conduction velocity (panel **B**), kaplan-meier estimates recurrence-free survival at 24 months of follow-up*.* Panel **(A)**: Average atrial voltage (cut-off ∼0.8 mV)*.* Panel **(B)**: Average conduction velocity (<0.6 m/s) (red line), average conduction velocity (>0.6 m/s) (green line).

Based on the difference between the three clusters, we finally assessed the individual prognostic significance of atrial CV and voltage for AF recurrence based on cut-off points of 0.60 m/s for CV and 0.82 mV for voltage. The analysis over a 24-month follow-up period revealed a substantial impact of CV on AF recurrence (Figure 8). At the 12-month interval, the incidence of AF recurrence was markedly lower in patients with normal CV, with over 90% remaining arrhythmia-free. In contrast, only approximately 70% of patients with reduced CV were recurrence-free. This trend continued through the 24-month follow-up, with 75% of patients with normal CV avoiding recurrence, whereas the arrhythmia-free rate in the low-CV cohort was reduced to 50%. Notably, the atrial voltage did not demonstrate a significant predictive capacity for AF recurrence during the observation period (Figure 9).

## 4 Discussion

Our observational study investigated the relationship between left atrial CV and atrial voltage in patients with initial AF ablation. The study's salient findings hinge on the marked differences in bipolar voltage parameters and CV between healthy individuals and patients diagnosed with AF. In particular, we identified significantly lower voltage and CV between control subjects and AF patients and a modest positive correlation between global CV and average voltage. Based on this relationship between atrial voltage and CV, our study highlighted three distinct phenotypes characterised by different combinations of CV, atrial voltages, and clinical characteristics in patients with AF, revealing a significant association between CV but not atrial voltage and AF recurrence over a 24-month follow-up period. Together, these data underscore AF's heterogeneity and the multifaceted nature of this arrhythmia.

### 4.1 Left atrial voltage and AF recurrence

Several studies have examined the relationship between atrial voltage and AF recurrence. For example, Masuda et al. (20) investigated consecutive patients undergoing initial ablation for PAF. In their analysis, left atrial LVAs after PVI were observed in 15% of the patients. LVAs were independently associated with AF recurrence even after adjusting for other related factors. Other studies have similarly shown that LVA is associated with AF recurrence after ablation (21, 22), with a more significant extension of the low-voltage regions associated with an increased risk of arrhythmia recurrence (23).

Nevertheless, it is essential to highlight that the AF substrate may be more than a localised LVA. Other studies have demonstrated that atrial remodelling comprises structural and electrical changes in which lower voltage, slowed conduction, and complex (fractionated) signals are more prevalent in AF patients than controls (24, 25). Many of these substrate abnormalities are more prominent in PersAF than in PAF patients, suggesting that these changes may reflect progressive atrial remodelling (24).

Our study found pronounced voltage differences between control subjects and patients with AF, particularly those with persistent AF. However, the atrial voltage did not significantly predict AF recurrence during the follow-up period. Nevertheless, the lack of significant prediction of AF recurrence during the follow-up period by atrial voltage might not align with findings from other authors, potentially stemming from variations in methodology and sample size employed.

### 4.2 Three AF phenotypes and the role of slow CV

The elucidation of the three distinct phenotypes within the AF population is a finding that adds a new dimension to our understanding of AF. Each phenotype, characterised by specific CVs, voltages, and clinical characteristics, may reflect different underlying pathophysiological mechanisms. These three phenotypes may represent different stages of AF progression, with variations in CV and voltage reflecting the evolving nature of the disease. Cluster 0 may represent an earlier stage, while Clusters 1 and 2 may signify more advanced stages, with Cluster 1 being particularly intriguing due to its normal voltage but reduced conduction. The observation that Cluster 1 exhibits a reduced CV despite a normal voltage is noteworthy. This raises the possibility that CV may be a more sensitive or earlier disease marker than voltage, the more commonly used marker. Moreover, the observation that AF recurrence was higher in Cluster 1 may reflect a more vulnerable phenotype in which considerable viable myocardium (can contribute to AF reinitiation in the vulnerable substrate produced by relatively slow average CV.

Previous studies have emphasised the importance of conduction abnormalities in AF, and our observations are consistent with our understanding of the complex interplay between electrical and structural remodelling in AF. For example, Heida et al. (26) conducted a case-control study comparing CV in patients with and without AF. Their findings align with our observations of CV reduction in the AF group. Similarly, the impact of CV on AF recurrence that we identified in our cohort is consistent with the impact of decreased anterior left atrial CV as a predictor of AF recurrence observed by Ohguchi et al. (13), the accurate determination of intra-atrial CV by Van Schie et al. (27) and the prediction of AF recurrence after PVI by Kurata et al. (28). However, our study builds on the methodologies explored by Coveney et al. (29), who provided an overview of atrial CV mapping algorithms to categorise these observations into three distinct phenotypes, providing a more nuanced understanding of AF. Zheng et al. (12) compared CV in the right and left atria in patients with AF and controls. King et al.'s (30) comprehensive review of myocardial CV determinants provided valuable insights into the factors affecting CV. Our study builds on these insights by exploring how these factors manifest in the three distinct phenotypes.

The localised relationship between late gadolinium enhancement-cardiac magnetic resonance and CV found by Ali et al. (31) and the study of CV dynamics by Honarbakhsh et al. (32) may align with our observed phenotype in cluster 2.

Finally, Frontera et al.'s (33) evaluation of the progression of electrophysiological phenomena in AF, focusing on corridors of slow conduction, supports our findings on the different stages of disease progression.

### 4.3 Mechanisms and relevance for AF patients

Three distinct phenotypes within the AF population, characterised by specific conduction velocities, voltages, and clinical characteristics, may reflect different underlying pathophysiological mechanisms, which might create opportunities to identify unique diagnosis, treatment, and prognosis implications.

A phenotype characterised by normal voltage but reduced conduction may represent an early stage of AF progression. This intriguing finding suggests that CV might be a more sensitive and early disease marker preceding voltage changes. At the molecular level, this could indicate subtle alterations in the atrial substrate, such as a reduced expression or abnormal distribution of connexins (34), a family of transmembrane proteins in gap junctions between cardiac cells. Additionally, dysregulation of specific ion channels, such as sodium channels, may contribute to this phenotype, reflecting an early stage of the disease.

The second cluster, with reduced voltage and slow conduction, may represent a more advanced stage of AF. Here, both voltage and conduction are affected, possibly owing to structural remodelling, such as fibrosis or inflammation. Fibrotic tissue can impede electrical conduction, leading to a more pronounced disruption of the atrial substrate (35). Chronic inflammation might alter voltage and conduction through changes in ion channel function or gap junction connectivity (36, 37).

Variable voltage and conduction may represent a heterogeneous group with varying degrees of atrial remodelling and diverse modulating factors. The phenotype characterised by normal voltage and normal CV reflects the absence of substrate disease. Therefore, it is possible to speculate that these patients required the influence of modulating factors for AF to appear, which is consistent with the observation of paroxysmal AF in most of this cluster.

The variability in disease expression reflects a complex interplay of genetic, structural, and functional factors, leading to diverse manifestations of AF. Disorganisation of the myofibrillar architecture, known as myofibrillar disarray, might contribute to this complexity, leading to nonuniform conduction patterns (38).

The moderate positive correlation between the global CV and average voltage observed in our study adds another layer to this intricate picture. This coordinated behaviour could be a compensatory mechanism to maintain atrial function or a reflection of shared underlying pathophysiological processes such as loss of viable myocardium reducing both conduction velocity and atrial electrogram amplitude (39, 40).

Our survival analysis further emphasises the importance of understanding these phenotypes. The correlation between the CV and AF recurrence underscores the predictive value of this parameter. It might guide therapeutic decisions, such as the choice of medication, ablation strategies, or follow-up frequency, tailored to the individual patient's risk profile. Moreover, understanding the underlying molecular mechanisms of these phenotypes could pave the way for novel therapeutic strategies such as connexin modulation or ion channel blockers.

## 5 Limitations

This study has various limitations. First, this retrospective study was conducted at a single institution, and the number of patients enrolled was limited. However, this is a very selected population undergoing the first AF ablation, using high-density mapping to obtain a map with thousands of points, allowing a detailed analysis of LA voltage and CV. We studied patients with PAF and PersAF, which may skew the results. Still, both types of AF were analysed during sinus rhythm or atrial pacing and were individually compared to controls in dedicated sub-analyses. Another limitation involves the calculation of remodelling metrics, which requires manual segmentation and output analysis with specific software, potentially contributing to measurement variability. This issue required two investigators to revise the activation points and segmentation.

For this reason, we also only focused on global CV and voltage parameters, although it is likely that regional changes in these parameters are sufficient to promote AF. Our results provide a more robust quantification of the atrial substrate but may be insensitive to small regions with abnormal electrophysiological properties.

The semi-automatic exclusion of the left atrial appendage and PVs, without assessing conduction velocities at the PV-LA junctions or within the LAA, constitutes a methodological limitation. Finally, the systematic use of ECGs, 24-hour Holter monitoring and external events recorders to assess AF documentation could underestimate the true prevalence of AF recurrence, particularly for short asymptomatic episodes. Despite the abovementioned limitations, our results can potentially generate hypotheses in the atrial remodelling process and should be assessed further.

## 6 Conclusions

Our study provides valuable insights into the complex interplay between CV, voltage, and clinical characteristics in AF patients. Atrial voltage and CV analysis uncovered distinct phenotypes, with slow atrial CV emerging as a significant predictor of AF recurrence, surpassing the predictive potential of atrial voltage. Identifying these different phenotypes and the prognostic role of CV may offer promising avenues for future personalised treatment and improved patient outcomes in AF. Further research with larger sample sizes and diverse populations must validate and extend these findings.

## Data Availability

The raw data supporting the conclusions of this article will be made available by the authors, without undue reservation.
